# Room for resilience: a qualitative study about accountability mechanisms in the relation between work-as-done (WAD) and work-as-imagined (WAI) in hospitals

**DOI:** 10.1186/s12913-023-10035-3

**Published:** 2023-09-30

**Authors:** Jan-Willem Weenink, Jaco Tresfon, Iris van de Voort, Teyler van Muijden, Jaap Hamming, Roland Bal

**Affiliations:** 1 Erasmus School of Health Policy & Management, Erasmus University Rotterdam, Rotterdam, the Netherlands; 2 Leiden University Medical Center, Leiden, the Netherlands

**Keywords:** Resilience, Safety-II, Healthcare teams, Hospitals, Accountability, Quality improvement

## Abstract

**Background:**

Central to Safety-II is promoting resilience of healthcare practices. In the “Room for Resilience” research project we focus on the role of horizontal and vertical accountability in healthcare teams and aim to discover how the relation between the two impacts team reflections and discussions. In this article, we report on an explorative study at the start of the project which aimed to assess the structures and dynamics of horizontal and vertical accountability.

**Methods:**

A qualitative study in six teams in three hospitals in the Netherlands. For the project, each team selected a specific clinical process to work on (e.g. pain assessment). We interviewed healthcare professionals, managers, and quality advisors about these processes, how they are discussed in practice and how teams need to account for them. Additionally, we observed the processes and how teams discuss them in practice. In total, we conducted 35 interviews and 67.5 h of observation. Transcripts and field notes were analyzed using thematic analysis.

**Results:**

Professionals at times varied in what they considered the right approach in the clinical process, with differing views on the importance of certain actions. When processes were discussed, this mostly was done during clinical work, and it often concerned reflections about the care for a specific patient instead of reflecting on the team’s general approach of the clinical process. Organized reflections on the processes were sparse. How processes were conducted in practice deviated from guidelines, mainly due to staff shortages, a perceived lack of value of a guideline, equipment issues, and collaboration issues. For most processes, accountability to hierarchical layers consisted of quality indicator scores. Professionals were tasked with registering indicator data but did not find this meaningful for their work.

**Conclusions:**

The observed different perspectives within teams on what good quality care is show the importance of having team reflections about these processes. How vertical accountability was organized at times impacted the conditions for teams to discuss resilient performance. Following these findings, we recommend that reflection on resilient practice and the role of accountability processes is organized on all levels in (and outside) the organization.

**Supplementary Information:**

The online version contains supplementary material available at 10.1186/s12913-023-10035-3.

## Background

Safety-II is considered a new paradigm of safety and improvement in healthcare [1]. It has come from a belief that traditional approaches that focus on preventing and addressing risks and harms (Safety-I) are insufficient in understanding and further improving patient safety [2]. Safety-II comes from the recognition that healthcare is a complex adaptive system and that acceptable and unacceptable outcomes in healthcare are both the result of performance variability in individuals and systems [2, 3]. Safety-II has found its way from theory to practice and is now included in national safety policies [4]. Central to Safety-II is the concept of resilience, which is increasingly considered a key factor in sustaining and improving patient safety [2, 5, 6].

There is a myriad of conceptualizations of resilience. This is partly due to the range of disciplines that have applied resilience in theory and practice [7–9]. To add to the multitude of interpretations and conceptualizations, resilience may be applied to individuals, teams, organizations and systems [10]. Most literature on resilience in healthcare however, has focused on the work being done by healthcare professionals, the so-called sharp end [10]. Less is known about the influence of actors and processes further away from the work, the so-called blunt end. Previous studies have suggested that more research is needed into contextual, structural, and relational details of how adaptive capacity is unfolding on multiple system levels, and how these contribute to resilience and responsiveness in healthcare systems [11].The concept of accountability might be helpful in studying this, as it captures the relational dynamics within and between organizational layers [12]. It can be used to demonstrate the way individuals, teams and healthcare providers account for their performance, which in turn may impact and explain how they work and are able to adjust to specific situations. In operationalizing resilience through the concept of accountability, we define resilience as “the capacity to adapt to challenges and changes at different system levels, to maintain high quality care” [13].

Accountability can be seen as ‘a relationship in which an actor feels an obligation to explain and to justify conduct to someone else’ [14]. Two types of accountabilities can be distinguished, horizontal accountability and vertical accountability [15, 16]. Horizontal accountability refers to how actors on the same hierarchical level – for example nurses in a team – account for their actions to each other, whereas vertical accountability refers to how actors account for their actions to higher hierarchical levels, for example how nurses account for their work to managers or how hospitals account to external regulators [17].

Horizontal accountability is about how people communicate, solve problems, and build accountability for positive outcomes. It creates trust, whereas when there is little or no horizontal accountability in an organization, people tend to engage in blaming and avoiding conflict [18, 19]. Horizontal accountability is an important concept to study in relation to resilience, as key processes of resilience concern activities of communication, collaboration, and learning and development through reflexive practice [13]. These processes are at the core of effective team interactions [20]. Good communication is a crucial aspect of resilient team performance, as it is essential for finding out what challenges must be faced and what resources are available to adapt to these challenges [21, 22]. Reflection is an important aspect of learning behaviour within teams that can improve how work is done and promote effective teamwork in the future [23, 24]. For this, it is important that team members experience psychological safety, so they dare to discuss concerns, share ideas, remain self-critical and reflect on challenging conditions in the past, present and future without the fear of rejection or blame [25]. Ultimately, the benefit of teams with a high psychological safety and established reflective practices is twofold: such teams will 1) be better equipped to adapt to emerging challenges and 2) will be better equipped to learn or draw lessons from adapting to these challenges to further improve.

Vertical accountability in healthcare is often built on Safety-I principles and designed from a work-as-imagined (WAI) perspective on healthcare [26]. Audit criteria set and inspected by external regulators and institutions are an example, or guidelines translated toward protocols and departmental work agreements. What healthcare professionals actually do to make sure all challenges and changes are met can be divergent on a daily basis, the work-as-done (WAD) [27]. Consequently, the concerns and impracticalities met in practice might not resonate well with how healthcare professionals need to account for their work to higher levels in healthcare. While this might lead to frustration and distrust upwardly in the organisation, it could possibly also disrupt horizontal accountability processes and reflection on current standards in teams.

In the “Room for Resilience” research project we focus on the role of horizontal and vertical accountability in healthcare teams and aim to discover how the relation between the two types of accountability impacts team reflections on concurrent practices and discussions of resilience. In this article, we report on an exploratory study that was conducted at the start of the project. In this exploratory study, we aim to investigate how healthcare professionals, team supervisors, managers and quality advisors view quality in terms of the carried out working activities (WAD) in relation to the general discourse and reflections surrounding quality instruments and performance (WAI). The objective was to assess the structures and dynamics of horizontal and vertical accountability prior to the improvement project and as a means to provide input to teams to work on safety improvement themselves. Teams will use these insights to work on improving a clinical process in the second phase of the “Room for Resilience” project.

## Methods

### Study design

This study is part of a larger action research project in which six healthcare teams in three hospitals in the Netherlands worked on improving a clinical process using a Safety-II perspective. Different teams and hospitals were included in the study to be able to distinguish team- and organization-specific from more general findings about accountability and resilience. We approached hospitals in our professional network of which we knew they were working on Safety-II or had identified it as a key area for quality improvement. Teams were selected in consultation with quality advisors from the three hospitals, based on their motivation for working on Safety-II and whether the project would fit in with their work and schedule. Each team selected a specific clinical process to work on, based on their perceived improvement potential or because they felt there were issues regarding accountability. Table 1 describes the teams and their clinical processes.

**Table 1:** Teams and their clinical processes (H = hospital)

Team and clinical process	Context	Background
1. Obstetrics and maternity wards (H1): medication verification process	Covering the outpatient clinic, acute and planned admissions, or discharges, physicians, nurses and obstetricians on the wards work interdependently to monitor and verify prescribed medication with current treatment plans to prevent drug interactions and mis-dosages	The ward reported to struggle with the verification process, resulting from a mix of a generally young and healthy patient group and admission of acute cases in which professionals prioritized providing acute care over verifying medication
2. Neurology and neurosurgery ward (H1): Pressure ulcers	Nurses try to prevent pressure ulcers from arising by repositioning patients and being alert on emerging bedsores. When wounds do occur, the nurses make a treatment plan in consultation with the wound care nurse	The neurological and neurosurgical patients are especially prone to developing pressure ulcers due to old age, malnutrition, immobilization, prescribed bed rest or comorbidity
3. Operating rooms (H2): instrument counts	To prevent unintentional retention of instruments and sponges in surgical wounds, operating staff counts all pre-listed instruments before the start of procedure and recounts instruments and sponges again before/at the onset of wound closure	As part of safe surgery, the department installed a protocol for counting instruments following two incidents. Since then, the hospital merged with another local hospital. Both locations kept their surgical units and professionals worked at both locations. Due to different histories with incidents and cultures, uptake and adherence to the protocol by the other location was limited
4. Emergency department (H2): transfer to acute short stay unit	To decrease patient processing time at the ED, an acute short stay (ASS) unit was implemented. Incoming patients that were considered stable and low-complex, but had to await test results, would be transferred to a more comfortable ASS whilst creating space for new incoming patients	The ASS unit was built in a separate location in the hospital compared to the other ED units. Although not far in distance, nurses and physicians take the distance into account when considering a patient transfer. Other factors influence patient transfer too, such as physicians having limited trust in the capabilities of ASS nurses due to their lower educational status than ED nurses
5. Vascular surgery ward (H3): early warning scores and pain re-assessment	Early warning scores are used by nurses to quickly determine acute illness in patients according to routinely scored physiological measurements at the bedside. When a threshold score is reached, the nurse informs the physicians and takes further action. The score has to be reassessed within two hours In pain assessment, the nurse asks a patient how much pain they experience on a scale of 0–10. If significant pain is present, i.e. a pain score of 4 or higher, pain medication is administered, followed by a re-assessment within the hour	Pain scores and revaluations have been introduced to prevent patients from experiencing pain needlessly. Early warning scores are tools to aid quick recognition of clinically deteriorating patients. The ward however struggled with full compliance in the digital pain reassessment form and the early warning score form
6. Gastroenterology ward (H3): Parenteral drug preparation and administration	In preparing and administering parenteral drugs, nurses use a four-eyes principle. By checking each other’s medication preparation before administration, errors may be prevented	Double checking with parenteral drugs is part of safe drug administration in nursing. The ward however remained to struggle with full compliance for all prepared and given parenteral drugs and dosages

### Data collection

Data collection comprised of interviews and observations. We conducted interviews with involved healthcare professionals from the teams, supervisors, managers, and quality advisors. Participants were selected in consultation with the involved quality advisor of the hospital and team supervisors and were informed about the research through an informed consent letter. Researchers did not have a professional or personal relationship with interviewees. A topic list (Appendix 1) was developed which focused on three main topics: 1) how the clinical process is being done in practice, 2) how teams discuss, reflect on, and try to improve the clinical process (horizontal accountability), and 3) how teams account for the clinical process to higher levels in the organization (vertical accountability). In total, we conducted 35 interviews across six teams and upper management layers of the three hospitals. Interviews were conducted in Dutch, recorded and transcribed verbatim. Selected quotes for this publication were translated into English.

Observations were conducted in concert with the interviews to better understand the clinical processes, how they are being done in practice (WAD), and if and how healthcare professionals reflect on these processes in practice. Observations were done non-participatory by shadowing professionals during working hours. After each observation, a report of the observation with reflections from the researcher was made. In total, we conducted 67,5 h of observations.

Data collection was conducted by four researchers experienced in qualitative research (JWW, JT, IvdV, TvM) and different scientific backgrounds (health services research, psychology, organizational learning). Data was collected between September 2021 and July 2022 during the first two months of acquaintance with the teams before commencing the improvement project. Prior and during data collection we held fortnightly meetings to reflect on the process of data collection and share experiences. Preliminary findings of the interviews and observations were discussed with the teams as a member-check and to determine strategies for reflection and learning as next steps in the project. Table 2 provides an overview of collected data in the teams.

**Table 2:** Interviewed participants

Team (researcher)	Interviews	Observations
1. Obstetrics (JWW, JT)	6	7,5h
2. Neurology and neurosurgery ward (JWW, JT)	6	9,5h
3. Operating rooms (IvdV)	4	18,5h
4. Emergency department (IvdV)	7	15h
5. Vascular surgery (TvM)	6	6h
6. Gastroenterology (TvM)	6	11h

### Analysis

All data were thematically analysed to identify overarching themes and patterns in the data [28–30]. First, four researchers read the interview transcripts and observation reports and made notes, after which these were discussed with all researchers to come to a code tree based on preliminary themes. These preliminary themes focused on discussion about quality between professionals, different perspectives, differences between work-as-done and work-as-imagined, the role of vertical accountability, vertical accountability in practice, the role of the organizational context, and resilient practices. Transcripts and reports were then analysed by four researchers using the code tree and afterwards discussed with all researchers to come to a finalized set of themes in the data. This led to three main themes in our data.

### Ethics

The Research Ethics Review Committee (RERC) of the Erasmus School of Health Policy & Management concluded that the research proposal sufficiently respected the safety and rights of participants and recognized the responsibilities of the researchers involved, and as such approved the study (ETH2021-0121).

## Results

We present our results according to three main themes: 1) presence of horizontal accountability in teams 2) vertical accountability processes, and 3) consequences for quality improvement. A summary of the thematic analysis for each team is provided in Appendix 2.

### Presence of horizontal accountability in teams

When analysing horizontal accountability mechanisms in teams, a few things stood out. First, there were few formatted or systematically organized discussions about quality on the six clinical processes. Second, health professionals looked differently at the processes and what is the right approach to take. Consequentially, different working methods were present within the clinical processes. Finally, interpretations of WAI and WAD differed between involved professionals. These were not only linked to simple normative causes but also to more fundamental considerations inherent to the care process or the work environment.

#### Talking about quality

Discussion of and reflection on the clinical processes by sharp end health professionals mainly took place regarding individual patient treatment as part of the primary process. Asking a colleague for advice, pointing out lapses and discussing what should be done next, as well as keeping each other informed and giving feedback, were essential elements of daily patient treatment. This way, health professionals keep each other in the loop about the current status of care processes and stimulate learning in less experienced colleagues. For instance, patients who have a high risk of developing pressure ulcers yet do not want to have their position changed, are deliberated among nurses and reported between shifts:

“No, it is more with direct colleagues with whom you provide that care. You pass it on to the other colleagues, and you describe it in the patient record. You write down like, “in consultation”, in the activity plan. You show that it is not a position change every 3 hours, you know, that there’s an aspect that makes it a little more problematic. But that way we do talk about it, it does get passed on. It doesn’t stay with the professionals attending at that time.” Participant #7 H1

When discussions occurred separate from daily activities, these primarily took the form of case reviews or discussions based on impactful incidents. These discussions often were unstructured and situated in the hallway or during end of day evaluation. For the most part, teams did not engage in structurally organized talks about practice standards or separate team reflection sessions regarding the state of the quality of care or how the team approaches or conducts the clinical process in general. As one health professional mentioned concerning whether to count instruments, discussions are not held recurrently but only temporarily surface after an incident:

“In the break room, we do not really discuss instrument counts. If an incident has taken place, we talk about it for a little while. But it's not that we discuss this daily, weekly, monthly or anything.” Participant #2 H2

#### Different perspectives on quality between healthcare professionals

Different opinions about what quality of care entails and how it should be achieved existed. Such differences were related to whom has which share within the processes (for example between different professions), distribution of responsibilities, opposing priorities or the added value of quality instruments such as protocols and registrations. Questioning the merits of the quality instruments like a protocol or registration form happened throughout the teams. While teams unequivocally named the shortcomings in the quality instruments in relation to carrying out the clinical processes, in some teams, healthcare professionals disagreed if strict adherence to the instruments was ultimately preferred or conversely not possible at all. For some, the quality instruments were seen as just a convenient additional tool to normal clinical skills, while others viewed them as nonsensical, being an insult toward professional identity or creating a false sense of safety.

“I think that we should do those checks. It's a nice feeling that you have a basis to start with. In addition, you also have your clinical view, don't you. It's not just about the controls. I mean all the checks can be really good yet someone can lay half dead in bed.” Participant #6 H3

Consequently, working methods also differed within the teams, relating to for example the amount of experience or the feeling of a sense of control. While inexperienced healthcare professionals argued that the quality instruments provide a useful handhold and thus used them more often, more experienced healthcare professionals often agreed that clinical skill and the patient inform the consideration between sticking to the quality instruments or using them more pragmatically. In some processes, healthcare professionals structurally deviated from the prescribed work practices since aspects were found redundant or inappropriate, while new colleagues had to find out about these local adaptions on the job. A health professional explained about double checking medication:

“We are a little more relaxed about that. You notice new colleagues who do not belong to the team, they really come after you [when the assessment is not done]. And then I try to put myself in their shoes. Look, we have agreed with each other, if you are busy, we will do it later. But I also understand them. They just got here, first department, and so they follow up on this. And then I also think, don't be so difficult, this will also be possible later. But on the other hand, I also have to lead by example.” Participant #3 H3

#### Differences between WAI and WAD

The gap between WAI and WAD had different reasons. Practical reasons were found in missing and ill-functioning equipment, or limited availability of time and colleagues causing extra work when trying to follow the ‘imagined’ work process. The suboptimal design of WAI in some quality instruments within the electronic patient records system significantly discouraged their use or made it challenging clarifying their usefulness to patients. Also, the mutual dependencies in some clinical processes made it hard to work conform protocol when a colleague took another route. On the possibility of re-evaluating early warning scores within 2 h as demanded by the system, a health professional stated:

“In terms of being compliant, it is more about, something came up, so you didn't make it within those two hours. It became two hours and fifteen minutes. Then the computer already says: you’re out. Or when the patient went for an examination, since he is so sick. The early warning score is bad and so he goes to the scan. He comes back from the scan, you do your checks again, but then you are three hours later. Then the computer says: failed. Yet the patient just went through the scan.” Participant #9 H3

Normative reasons were also given for WAI and WAD differences. A sceptical stance toward the benefits for the patient, a focus on prevention or an absence of perceived safety risks made healthcare professionals feel that strictly following WAI is unnecessary or not useful.

“Right, I see my patient is in excruciating pain. I'm not going to spend 10 hours looking for my colleague. I want my patient to be pain free, that's my goal, that's why I became a nurse, I want to help.” Participant #2 H3

Besides these normative causes, more fundamental considerations inherent to the care processes could be attributed to WAI and WAD misalignment. Unpredictability during surgery, acutely admitted patients or handling in the interest of patient treatment in ward care place a nuance on working strictly compliant and made priorities shift. Healthcare professionals in these clinical processes accounted for these surprises by making trade-offs in directing their time and effort, anticipating on possible unwanted surprises or unnecessary actions in the interest of the clinical process or the patient.

“We also have situations where people are admitted acutely. So, someone is giving birth at home and things are not going well and then are sent in. They come to the hospital, and everything has to be done. Well then you understand that we are not going to ask for the medication verification first. But then, when will we do it? And then it is forgotten because that patient has already given birth and will go home tomorrow. Big deal, or whatever.” Participant #4 H1

### Vertical accountability processes

When exploring vertical accountability regarding the clinical processes, we noticed that this was structured in several ways. Additionally, the role of managers and their perspective were important to understand the way health professionals need to account for clinical care. Finally, we observed different ways of how vertical accountability impacted the work of health professionals.

#### How vertical accountability processes are structured and organized

Differences existed in how teams had to account for the clinical processes to higher levels in the organization. For almost all processes, data needed to be registered that served as input for indicator scores or other quantitative quality information. These indicator scores had set criteria, resulting in, for example, coloured results (green, orange, red) or sufficient and insufficient assessments, and are a topic of discussion between team management and higher hierarchical layers in the hospital. Indicator scores predominantly concerned whether a process or activity had been registered and was seldom linked to actual clinical outcomes.

“Because I do think that we have quite some indicators that look at the process and not so much at the outcomes. Though you’d prefer to look at the outcome and then look back like: could we have seen this? Does a registration process really help us with that or not? But in practice it actually went the other way around: if you just register this, it will help us to get less pressure ulcers later. And that’s questionable. That is very much questionable.” Participant #1 H1

Indicator scores were mainly calculated on a team or department level. For the process of registering early warning scores and pain assessment though, indicator scores were calculated on an individual level, in which insufficient scores resulted in a ‘talk’ about improvement with the specific professional. Instead of focusing on quality improvement of the team, it focused on singling out the ‘cause’ of low indicator scores. Team management in this instance realized that this might lead to unpleasant situations. The choice for individualizing these scores seemed to come from a sense of hopelessness and unsatisfying experiences with targeting the whole team. In other teams, management purposefully did not single out individuals, but targeted improvement on a team level.

“This morning A. was busy, because they couldn't get the scores any further up, so they were really doing a bit of research in the electronic record system to see which professional has low scores. And that sounds very bad, but you just can't do anything else. Continuing to address the group does not seem to work, because it is ineffective for everyone who does a good job and it does not contribute to improvement. On the one hand, that’s the beauty of the system [that you can single out causes] and on the other hand it is quite a far-reaching measure. But we are responsible. So we have to do it on an individual level.” Participant #10 H3

#### Managers’ need for grip and control

Even though most managers had a clinical background, some realized they had been away from the floor for a while. This resulted in having difficulty in really understanding what happens in clinical work and for what reason, and at times in keeping up with the latest professional developments in taking care of patients.

“Well, the focus is much more on management and less on content. And we do try, at least, I'm talking about myself, I notice that I do no longer follow all developments in the workplace and clinical work. But I do trace whether we are still well-organised and what the quality of our care is. Of course, you have a lot of conversations about: how are things going in your department.” Participant #1 H1

For managers, to have a grip on quality and safety is important, making sure they have potential risks for patient safety in view. Between teams, managers had different experiences with whether they had a clear view on the quality and safety of the clinical process. Indicators designed from a WAI-perspective function as a tool to grasp this, but in practice then become a synonym for high quality. So instead of being just ‘an indication’ of whether quality is good or bad, it becomes the assessment of whether quality is sufficient. As a quality advisor wondered:

A., a quality advisor, now indicates that these scores do not say much and are separate from good care. But she does wonder how management can focus on quality without those percentages. [observation notes H3]

#### Perceived impact of vertical accountability processes

The need to have a grip on quality might result in asking for as much quality information as possible, instead of a focused query of specific information. What comes into play here as well is that managers often need to account to their internal supervisors (e.g. hospital CEO) or external regulators, in which they have to show that they are in control and, in case of insufficient indicator scores, are doing the right things to improve. In some cases, this resulted in a feeling of professionals that they are doing things for an accreditation organization, health insurer or regulator. It might leave a false sense of safety, instead of really being convinced that the quality and safety benefits from strictly following protocol or registering data.

“In practice we work differently. Whenever we think this is a patient who does not mobilize, gets little or poor nutrition and is also not resilient, we use a specific mattress. And we almost never have pressure ulcers, so I'm fine with that. In that vertical accountability, based on the reports submitted, I was told that I was using the beds for the wrong patients. I used the more expensive beds for patients to prevent pressure ulcers instead of letting them develop pressure ulcers first and then giving them the right bed to counter ulcers.” Participant #5 H1

There was not only a perceived impact of the presence of vertical accountability; the quote above shows that vertical accountability can also be misaligned with the normative orientation of the care workers. Regarding instrument counts, team members experienced rather a lack of vertical accountability as team leaders did not need to account for this specific process to higher levels in the organization. It resulted in some professionals experiencing instrument counts not to be of high priority. This does not mean that professionals want management to get actively involved in instrument counts, but they did indicate that they would appreciate it if management showed more interest in how the clinical process unfolds in practice. This preferred visibility of management and showing interest in the clinical work, is something that came back in multiple teams and could help managers to get a better view on what happens in patient care as well.

They (the nurses) talk about the people from above, who never come to the department, but who determine what happens. Another nurse says: ‘if she would just follow along for a day.’ They also never see a quality advisor from Quality & Safety. Nobody knows the names of the quality advisors. They indicate that sometimes someone comes to have a look and then says it has to be done this way. And then 5 minutes later, once that person is gone, the nurses do it their own way again. [observation notes H3]

### Consequences for quality improvement

From our data, it became evident that accountability may impact reflection and learning of teams and their resilient practices. At the same time, other aspects than accountability had an impact on reflection and learning as well. The design of quality structures directed discussions and efforts surrounding quality improvement, which in turn were influenced by contextual factors within and outside the organisation.

#### Accountability: a focus on registering indicators

We noticed a focus on registration of indicators instead of quality improvement. In these cases, the registration then becomes reality, meaning that if care activities or measurements are not registered, they are considered not done. This has consequences for the satisfaction and work experience of healthcare professionals and might lead to management not having a specific interest in how things are going and could improve, but solely focusing on that the numbers improve.

“A manager who steers for green checks [*i.e. who goes by the books and aims for the registration of indicator scores]* does well with the board of directors, but this is also what makes nurses dissatisfied. My main concern is nurse turnover, staff retention is the most important.“ Participant #12 H3

This also relates to the previous observation that in some instances there was a felt lack of guidance or involvement from vertical layers, which makes that quality improvement for the specific process is not considered a high priority by the team. As a result, healthcare professionals might focus on the registration when prompted, but are not stimulated to have discussions about the quality of their clinical work when managers are going by the books and aiming for the registration of indicator scores.

A part of this is also how indicator scores and ‘quality information’ is organized and fed back to teams. Performance was often not fed back to teams or did not meet their needs. If there was feedback from vertical layers, the feedback was at times based on incorrect numbers or too much focused on WAI and insufficiently in line with health professionals’ clinical work. This then resulted in discussions about the validity of numbers instead of reflections about the care behind these numbers and how they could contribute to quality improvement.

“Where do these numbers come from? I can’t follow it. And that irritates me because you are working on quality improvement in a constructive way and then you receive these scores, and you think ‘I think this is not possible at all because no people are admitted to the outpatient clinic’.” Participant #3 H1

#### Previous and ongoing attempts for quality improvement

Teams had previously worked on quality improvement on the selected processes, and this was almost always approached from a pragmatic perspective looking for practical solutions. Quality efforts on the nursing ward-level for example, were mostly centred around dedicated task groups made responsible for spending attention and effort at improving the clinical process. The initiatives deployed by these groups initially took off quite well, but after some time got bogged down and it seemed hard to keep initiatives on the agenda and in the minds of people. Similar observations were made in other teams, where energy and motivation to learn and reflect had dropped because of previous experiences, or the team struggles to keep momentum after a reflective meeting:

“We had that FRAM-analysis, we have written out a lot of processes. And then it falls silent again. Now, of course, we have the planned appointment next week, but still will we send those people a reminder saying 'hey, look at that flowchart to see if there are still things in it that you want or can do something with'? Because now you actually hear nothing. A number of people know that they have to do something with it and that things are not good yet. And some people are still very annoyed.” Participant #1 H2

#### Organizational context

What at times makes quality improvement difficult as well, are the organizational context and organizational mechanisms: for example, big teams without much social cohesion or professional interaction, when there is a history of intervention by an external regulator, or after a hospital merger when two different organizational cultures need to go together. Combined with budget cuts, the impact of COVID-19, a feeling that departments work independently on their quality and a top-down accountability approach from the blunt-end toward healthcare professionals, these contextual factors impact the felt urgency and believed significance in healthcare professionals and their direct superiors. One team leader indicated how a layered organization hinders the efficiency of decision making in quality improvement processes:

“Everyone wants to have their say about it. That can be quite annoying. Because then you have to wait for that one and then that one again. And if you have a small organization where one or two people decide, then it's done quickly. But now everyone has to agree with it. Everyone has to say something about it. And all that has waiting time. And you also have different divisions for whom it can be different. So if you just had a small organization, I think it would be much more efficient.” Participant #6 H1

## Discussion

In this study, we explored the role of horizontal and vertical accountability and the impact it has on how hospital teams view and discuss WAI and WAD. We found that horizontal accountability mechanisms are often related to the treatment of individual patients and less so to more general care processes within the team. Overall discussions moreover tend to be decoupled from actual work practices. Vertical accountability was often focused on the registration of indicators or keeping up with protocols and professionals did not experience this as contributing to quality improvement within teams. Consequently, how vertical accountability was organized at times impacted the conditions for teams to discuss resilient performance, yet other aspects such as organizational context, played a role as well. In the next section, we reflect on the methods and subsequently discuss the main findings of this study.

### Methodological reflection

A strength of this study is the variety of included teams and hospitals, which means that we can draw conclusions about accountability and resilience that go beyond a specific organizational context. By focusing on the layered nature of healthcare and including both sharp end and blunt end professionals in our interviews and observations, we add to the literature on resilience and Safety-II [11]. Another strength is the combination of interviews and observations in data collection, allowing us to check certain aspects in practice, and ask about certain observations in interviews. At same time, a limitation is that to be fully able to identify resilient practices, longer observations and structural embedding in the teams would be needed. We heard a lot about practice but were not always able to observe things we heard in interviews. Also, making use of group sensemaking would be beneficial for understanding the relational dynamics in the different processes and on multiple levels.

In interpreting the findings, it is important to consider certain contextual factors of the study. First, we chose to use accountability as a concept to study how teams work on quality improvement. Although we identified other relevant aspects that impacted this (i.e. previous attempts and organizational context), using other concepts could result in further insights. Second, we approached hospitals that had Safety-II on their agenda and selected teams that were motivated to work on Safety-II and had time to fit the project in their work. Working with less motivated or experienced teams and hospitals (in relation to Safety-II) could impact the findings. Additionally, the study only focused on the Netherlands. In other countries, vertical accountability processes regarding regulation and accreditation might be organized differently. The identified mechanisms and impact of such processes on horizontal accountability are most likely similar though, and as such of international relevance as well.

### Talking about quality in teams

Discussion about the clinical processes seemed to take place predominantly during direct patient treatment, limiting reflection to the individual patient level. At the same time, perspectives, working practices and their believed importance differed between healthcare professionals within the teams. For the most part, teams did not engage in team discussions separate from daily patient treatment as a means to share and discuss different beliefs and working methods within the clinical processes on a meta-level. Through sharing and recalibrating contemporary working practices, quality of care could potentially become more tangible, while it could also help new team members to learn and progress toward an explicit working standard.

Little guidance and attention were seen in the vertical accountability structures for having such reflective discussions. A missed opportunity, as literature suggests that teams that are able to share and reflect show great potential for innovation [23]. While often missed in popular research endeavors, studies of healthcare teams utilizing reflection resulting in improved team effectiveness exist [31]. For example, teams have demonstrated successful engagement in reflection in after-action reviews with positive effects on speaking-up behaviour across hierarchy structures [32], sharing experiences about patient and clinical cases positively impacting team building and effectiveness [33], and video-assisted performance debriefings [34] resulting in increased team performance and better adherence to best practice guidelines. Other interventions aimed at improving patient safety have conversely created a need for reflective meetings as a side-effect [35]. Such studies frequently touch upon the importance of open communication and knowledge sharing, highlighting the importance of psychological safety throughout the teams. While reflection arguably might stimulate a shift within the psychological safety of teams [32], the positive associations with (inter)professional collaboration and learning behaviour [36–38] cannot be omitted. Psychological safety can be an important aspect when considering a reflective practice and should not easily be neglected.

### Making indicator scores contribute to instead of hinder reflection and learning

Indicator scores dominated vertical accountability processes and were not only used as an indication for quality, but in fact have become the definition of quality [39, 40]. This often leads to discussions about the validity of numbers between the team and management, instead of reflections on provided care behind these numbers, and to health professionals feeling distanced to quality information in the organization. A focus on numbers without trying to understand the underlying story does not contribute to learning. This does not mean that indicator scores should be dismissed altogether, but it does require a different role of indicator scores in accounting for healthcare, namely as ‘tin openers’ instead of ‘controls’ [2]. As such, accountability might become generative in a sense that it will facilitate instead of hinder learning [41]. Narrative accountability processes that focus on the story behind the numbers, could provide tools to put this into practice [42]. It seems important that all those involved, such as professionals and managers, are included in designing these alternative accountability processes as this might lead to a better understanding of each other’s position and perspectives regarding accountability of care and a better alignment of vertical accountability with clinical work in teams.

A complicating factor is that accountability of care is not isolated to the relation between health professionals and team management. Accountability processes and quality improvement processes are influenced by external parties such as governmental regulators as well [43, 44]. We observed in our study that some health professionals felt that registering quality information was primarily done for external parties. This means that a shift towards generative accountability from a Safety-II perspective, also requires change from these external regulators. This change should be focused on providing structures and mechanisms that support resilience, for example by shifting regulatory focus from compliance to consistency and recoupling gaps between WAI and WAD [45].

### Organizing room for resilience

As a result of a persisting focus on indicator percentages as a synonym for quality, little time and effort was directed at stimulating quality talks on the work floor by vertical accountability processes within the three hospitals. Concurrently, meta-reflections about working practices don’t seem to emerge spontaneously within the studied teams. As a consequence, little room for discussing the value of resilience seems present within the six clinical processes. This is a missed opportunity for both horizontal and vertical accountability structures, as the concept of resilience might offer vantage points on the functioning of health systems on different levels [11]. To do so, not only a clear understanding of what objectives resilience aims to achieve is important, but also what resources are used when resilience is enacted [46].

Counterintuitively, vertical accountability structures in turn would need to be curious and supportive toward deviations from rigid quality instruments. The focus should be on appreciatively inquiring the professionals on their perceived and documented outcomes and their worries. Not only have such management approaches been argued to positively influence safety climates [47, 48], also more specific managerial and leadership strategies to support resilience have been formulated [49–51]. Since resilience can be difficult to grasp on multiple system levels and time scales, future research could benefit from frameworks accounting for such difficulties [52]. Even so, seeking to understand what restructuring and reorganization of resources and practices needs to be done to better suit resilience in hospital teams, should not be subjected to instrumentalising. Similar to the quality indicators, resilience frameworks should aid in the sensemaking of entrenched relationships and discussions between system levels, not interpreted as a golden standard of necessary requirements before resilience can be present in teams.

## Conclusion

The current study highlights important dynamics and mechanisms between different system levels. In horizontal accountability, different views, working methods and an absence of discussing quality on a team level seem important to address. In vertical accountability, the added value and current approach of quality instruments, as well as a lack of vertical accountability within the larger organisational context, need to be questioned. These aspects should be integrally accounted for when advancing quality work beyond just stating WAI-WAD differences or exploring expressions of resilience, as previous literature on Safety-II and resilience seems to suggest [53–55].

In the second phase of the “Room for Resilience” project, we aim to translate these findings through action research back to the teams and vertical accountability structures. Teams and managers will discuss findings and together choose a method or approach to work on quality according to Safety-II principles. Subsequently, teams and managers will explore together how resilience in the healthcare processes can be addressed best from multiple needs and perspectives. By bringing together healthcare professionals, team supervisors, managers and quality advisors in these processes and making these mechanisms explicit through (reflective) group discussions, we take a next step in creating and operationalising room for resilience.

### Supplementary Information

**Additional file 1:** **Appendix 1.** Topiclist teams.

**Additional file 2:** **Appendix 2.** Thematic analysis for each hospital team.

## Data Availability

Data is available upon reasonable request. All data are in Dutch.

## Abbreviations

CEO: Chief executive officer
WAD: Work-as-done
WAI: Work-as-imagined
