# Supplementary material for: Room for resilience: a qualitative study about accountability mechanisms in the relation between work-as-done (WAD) and work-as-imagined (WAI) in hospitals
Source: BMC Health Serv Res. 2023 Sep 30;23:1048. doi: 10.1186/s12913-023-10035-3 (PMC10543860; doi:10.1186/s12913-023-10035-3)
Supplement: Supplementary file 1 — Additional file 1: Appendix 1. Topiclist teams. [file 12913_2023_10035_MOESM1_ESM.docx]

**Appendix 1 Topiclist teams**

**Introduction**

**Function and roles**

- Can you tell us something about your position?
  - Professional background?
  - How long have you worked in the team/on the clinical process?

**Clinical process**

- What does the process look like?
  - What is being done by whom?
  - At what times?
- What are your experiences with the clinical process?
  - What is going well?
  - Are you running into something? Challenges in the process?
- To what extend do you feel your vision or perspective regarding the process differs from others?
  - Different perspectives/interests?

**Experiences with mutual feedback and reflection**

- How does the team learn/reflect/give feedback on what is going well? And on what goes less well?
  - With whom? (team, who are they?)
  - At what times; formal or informal?
  - Where?
- What space or time is there to address each other about what is going well/not so well?
  - Which connections/facilitate reflection/feedback
  - What is the influence of accountability?
- How do you view the importance of addressing each other?
  - Why is that important?

**Experience with hierarchical accountability for the clinical process**

- What are you accountable for with regard to the process?
  - To whom do you account?
  - When are you accountable?
- What is your experience with hierarchical accountability with regard to this process?
  - What is the influence of accountability on the process?
- What could be done better? How can more be in line with practice?

**Closing remarks**

- Do you have any questions/additions/comments?
