# Supplementary material for: Room for resilience: a qualitative study about accountability mechanisms in the relation between work-as-done (WAD) and work-as-imagined (WAI) in hospitals
Source: BMC Health Serv Res. 2023 Sep 30;23:1048. doi: 10.1186/s12913-023-10035-3 (PMC10543860; doi:10.1186/s12913-023-10035-3)
Supplement: Supplementary file 2 — Additional file 2: Appendix 2. Thematic analysis for each hospital team. [file 12913_2023_10035_MOESM2_ESM.docx]

**Appendix 2: Thematic analysis for each hospital team**

|  | **1. Horizontal accountability** | **2. Vertical accountability** | **3. Impact on reflection and learning** |
| --- | --- | --- | --- |
| 1. Obstetrics and maternity wards (H1): medication verification process | Prescriptions and orders are checked during admission, ward rounds and discharge, although it is often not considered a priority due to the healthy nature of the patient population.  Dedicated quality task group distributes reminders about importance of verification and has experimented with a daily question and reminder note from the nurses to the physician, but struggles to keep it on the hearts and minds of professionals. | Registration of the activity of medication verification. Two indicator scores represent the percentage of medication verification at admission (between 60 days prior to admission and one day after) and discharge (clinical conversion or discontinuation), as a proportion of total admissions. Registration leads to a coloured assessment (green = good, orange = average, red = insufficient), which is discussed between the ward management and the department head. | Dominant focus on improving numbers using quick-fixes within the electronic record system. Little to no reflection on the interdependencies, difficulties and varying priorities between disciplines in the verification process. |
| 2. Neurology and neurosurgery ward (H1): Pressure ulcers | Discussions happen on the individual patient level during daily care in the hallway, or during separate clinical lessons and case studies. Assessment of pressure ulcer risk is based on clinical experience and patient characteristics. Professionals initially focus on prevention of pressure ulcers. Nurses with dedicated experience in pressure ulcers work together in a task group.  Registrations of forms in the electronic patient records during nursing anamnesis are done variably (assessment) or sparsely (re-assessment). Protocol is sparsely used. | Two indicator scores measure percentage of admissions with screening within 4 hours of admission, and the percentage of timely daily repetitions in case of increased risk (as proportion of total admissions on the ward).  Indicators scores form the input for the talk about the state of pressure ulcer care on the ward between ward manager and department head. Focus on actions made by ward manager to improve the process.  Hospital-wide task groups focusses on stimulating registrations and standardisation through protocol adherence. | Learning takes place in the form of bedside teaching as part of the training period, but practices can vary between professionals.  Meta reflection about team practice standards is absent.  Difficulties and practices remain unknown to the larger organisation. Focus on prevention is not recognised as such. |
| 3. Operating rooms (H2): instrument counts | Opinions about the usefulness and feasibility of protocol adherence differed in the team, discussions were scattered and sparsely structurally organized. Preferred working methods varied between professionals. There was one working group tasked with facilitating easy use of protocol, yet different opinions were not represented in the working group (i.e. only pro-protocol) | Protocol specifies framework for instrument count procedure (e.g. four eyes principle, all instruments counted before wound closure), and counting lists are specified per medical speciality (e.g. neurosurgery, orthopaedics).  Protocol in place that prescribes the process of instrument counts. There are no felt consequences for not adhering to this protocol. Management strives for uniformity, particularly in light of external accreditation rounds end of the year. Where the head of department argues that OR-assistants should decide on that uniformity, team leaders interpret it as protocol adherence by all. Professionals perceive that management does not find the protocol important for quality and safety but feels that the protocol has been put in place for external regulators, as non-adherence is not penalized. | Discussion remains unsettled and uneasiness in some professionals continues to simmer as to what is expected from them as individuals and as a team. Little room for reflection, learning on the job remains tacit. |
| 4. Emergency department (H2): transfer to acute short stay unit (ASS) | A relatively new unit within ED, yet geographically dispersed from other ED units. ASS nurses rely on nurses and physicians from other ED units to transfer patients to the ASS unit. Written requirements for transfer are often ignored or stretched, particularly during busy or quit times, leading to crowded or unsafe situations at the ASS according to ASS nurses.  Although most ASS nurses understand the necessity to stretch requirements for transfer in busy times, they want to safeguard the quality and safety of such transfers and avoid a ‘back-and forth’ of patients as well. Discussions and evaluations of the quality and safety of transfers to the ASS unit *between* health care workers of different units are considered beneficial, but do not happen regularly due to conflicting shift changes. | Management monitors processing time of incoming patients on the ED and to what extent the ASS unit is used appropriately (i.e. for the right reasons) and in turn, actually improves processing time of patients at the ED. While physicians control the flow of patients between the ED and ASS, managers have to account for the processing times.  Management signals problems with processing time to the Board of Directors, but does not communicate these stats to the ASS unit. Professionals are not held accountable for processing time.  Due to COVID and turnover in management there have been very few organized team meetings, with no to limited time to address quality issues. To some extent this has translated to demotivation to work on quality and safety within the ASS unit.  ASS nurses have expressed their wish to management to be more visible on their unit, so they can see and understand their experienced (quality) issues and to lower the threshold to signal quality issues to management. | Hardly any reflection, let alone learning, on the ASS unit due to a lack of organized or structural meetings to discuss quality and safety within and between ED units on the topic of transfer to ASS unit. Also a lack of communication on stats of processing time and transfer to ASS unit between management and professionals. |
| 5. Vascular surgery ward (H3): early warning scores and pain re-assessment | Professionals are critical toward the instruments and/ or see it as an additional tool. Favour to rely on clinical view and communication with patient for pain assessment, or deliberation with fellow nurse for early warning scores.  Re-assessment for both processes seldom registered and registration feels redundant or is hard to achieve according to professionals. | Strongly influenced by accreditation standards, percentages of registered re-assessments as a proportion of total admissions are prime performance indicators of both processes. Percentages are only available higher up in the hierarchy.  At the same time, managers and team leaders question if improving percentages will benefit the patient. | When scores drops, needed improvement in percentages is addressed during management/team meetings. Also, professionals can be questioned individually during quarterly meetings.  Reflection and learning about perceptions and difficulties on the working floor is missing in the accountability structure. |
| 6. Gastroenterology ward (H3): Parenteral drug preparation and administration | Doubling checking is done with uncommon medication, but less so with common medication. Work practices differ between experienced and novice nurses. Feedback about slips and lapses is given during shifts. A dedicated taskgroup is made responsible for medication on the ward. | Out of fear of interventions by external regulators, compliance driven focus on registrations of medication administration. Although it is felt that individual healthcare professionals are responsible, management and board feels ultimately responsible. Discussions focus one-directional on making the process as intended. Felt distance between sharp- and blunt end is large. | No reflection on different practices or realistic care standards, or the reasons why registrations don’t add up (e.g. defaulting equipment, understaffing).  New and inexperienced colleagues need to learn work practices on the job. No feedback from organization on registrations. |
